# The Spectrum of the Crimson Tide

**Published:** 1867-12

**Authors:** Rufus King Browne


DENTAL REGISTER.

Vol. XXI.] DECEMBER, 1867. [No. 12.

### Original Communications.

### THE SPECTRUM OF THE CRIMSON TIDE.

BY RUFUS KING BROWNE, M. D.

If Newton put our sun and the planets in a scale and accurately estimated their weight, so the discoverers of spectral analysis, by *means* of the light of both sun, planets and stars placed them in a sure testing tube and analyzed them, ascertaining their constitution, their state of aggregation, and nearly completely their chemical composition, with the same certainty with which we analyze in a crucible, a fragment of the crust of our earth. The one was a vast physical problem solved, the other a vast chemical revelation.

Almost everybody who has learned the meaning of the name knows the wonders that spectral analysis has accomplished, in the varieties and states of matter, near to, and most remote from us.

It has *shown* the world the actual existence of burning stars, those apparently just kindling, flashing, and extinguishing bodies. Astronomy was forced to suspect more than existed; and has ascertained the chemical character of their conflagrating substance.

This it does because the brilliancy of such a substance, or any substance, in such a vaporous state will present to the view transmitted through a prism, a three-cornered piece of transparent polished glass, a telescopic lens, of which two the spectroscope consists—one or several colored lines—which appear only (each one or several) from the light of each substance.

But it has achieved far greater wonders with the matters within us, the changing and transforming substances of our flesh and blood. But what is most marvellous is, that in this field of employment it has met with substances far more delicate in their powers of revelation than itself, that is to say, substances without the intervention of which, the spectroscope itself would fall far short of detecting in such infinitesimal quantity. Let us briefly narrate the story: Rushing in all the minute blood channels, which in great part constitute our fleshy structure, frequently at a velocity which no mass of either living or inert matter, either least or greatest in nature has yet equalled*—mingling with the amber colored stream of liquid of the blood, are certain minute reddish, rounded, soft solid bodies, the blood-red corpuscles.

High powers of the microscope, in the hands of the most skilled observers reveal, that scarcely any two of these *are of precisely the same size*, some of them are from five to six times the size of others. It is agreed that in human beings, their average length is about the 1-2400 of an inch. It would be possible if they were closely packed together, for 8,126,464 to lie in a space occupied by a pin's head. The tiny red drop which issues from the puncture of living flesh, by a prick of a needle, consists of about 5,000,000 of these bodies; and a room sixty feet long, thirty feet wide and fifteen feet high, could not contain so many grains of corn as there are red corpuscles in a single teaspoonful of human blood.

The chief and peculiar office of these little bodies has been

*I have calculated that the blood-red corpuscle moves four hundred times its own length in a second.

long suspected, by the circumstance of their regularly undergoing two changes of color during their round of the circulation. One change from a purple hue to a scarlet, and another change from the latter hue to the former. These little bodies are the carriers of oxygen from the lungs, where they take it from the air, throughout the body. During its passage through the blood vessels of the lungs the blood expels carbonic acid, and appropriates oxygen. This oxygen it is now known is taken up by the red corpuscles. Ever since this fact was discovered it has been assumed by physiologists that the coloring matter of the corpuscles was capable of combining with oxygen in the lungs, and of afterward giving out that oxygen again in small increments, as it were—to the substances surrounding the blood vessels—*i. e.* the tissues. Spectral analysis gives us the perfect demonstration of the fact.

Years ago, somebody recorded the curious fact, that when a ray of white light passes through a *solution* of blood, and is then passed through a prism, *two* dark bands make their appearance in the green color part of the spectrum. Lately a distinguished English physicist verified and repeated the fact, but in his hands it became the initial step of a new train of research.

This observer treated a solution of red blood corpuscles with a "reducing" agent, that is, an agent which steals away the oxygen from the reduced substance, and observed the color of the solution. It almost instantly changed from the color of the red corpuscles of arterial blood, scarlet, to purple red, the hue of venous blood. On examining the spectrum of this, by means of the spectroscope, he observed that the two dark lines had disappeared, and that only a single line intermediate in position between them, was visible. On shaking a part of the solution of red corpuscles in a tube with air, the scarlet hue returned; and when again examined by the spectroscope the two lines in the spectrum, characteristic of the scarlet colored substance re-appeared; but these again after a few minutes disappeared, and the solution showed by the spectroscope, the one line characteristic of the now purple hued substance of the solution of red blood corpuscles.

The spectroscope thus demonstrated that the scarlet arterial blood lost its oxygen in the first instance, to the reducing or deoxidizing agent, and subsequently appropriated oxygen again, from the air when shaken in it.

This, to physiology immensely important, because truly demonstrated conclusion, was thus stated by the discoverer:

"The coloring matter of blood, (of its red corpuscles) is capable of existing in two states of oxidation, distinguishable by a difference of color, and a fundamental difference in the action on the spectrum. It may be made to pass from the *more* to the *less* oxidized state, by the action of reducing agents, and recovers its oxygen from the air."

But even more wonderful, physiologically considered, is an unnarrated fact, which has not yet traveled beyond the private records of observation. This is the fact, that these red globules, are not, as is universally believed, carried by the fluid as impelled by successive contractions, from the heart, but move through the liquid blood, at a much faster rate than the liquid itself. Each globule may, therefore, move at a rate different from time to time, and different from its fellows, although in general terms, they concur or move together at a certain rate. Upon the perception of this fact, no doubt, will turn many future discoveries of the condition of varying states of health and disease. Mankind have always had a dim instinct, hitherto uncorrected and unsupported by science, that many states of disease are dependent on the blood.

May it indeed, turn out to be at least scientifically true, that "*The life is the blood.*"

In these observations, there was a perfect demonstration that this coloring matter, constituting the distinctive matter of the red corpuscles, named *cruorine*, could easily pass from one state to the other, and the reverse.

In the more oxidized—the scarlet state—that in which it is found giving, by the corpuscles, to the arterial blood, its scarlet hue, it is distinguished as *scarlet cruorine*, and in its reduced or less oxidized state, that in which the red blood corpuscles give to venous blood its purple hue, it is known as *purple cruorine.*

It is hardly necessary to designate what a consummate explanation these facts afford, of the oxygen appropriating and carrying capacity of the red blood corpuscles, nor what a soul inspiring exemplification it is of the achievements of spectral analysis.

In the lungs the purple cruorine of the red corpuscles of venous blood, appropriates the oxygen from the atmosphere, and becomes *scarlet* or arterial cruorine; and in the whole of the general circulation—in the minute blood channels, this cruorine of the red globules having passed through the arterial part of the circuit, loses a part of its oxygen and passes back to the purple or venous state.

But those results, high though they be, have been exceeded in direct practical consequence, to the world at large, by those achieved with the micro spectroscope.

An eminent London optician, Mr. Lorby, has in inventing and using it, supplied medical jurisprudence with a new and certain means of identifying the character and variety of dried blood stains. By it a scrap of blood-stained fabric, 1-10 of an inch square, containing possibly, not more than 1-1000 of a grain of red corpuscle coloring matter, may be ascertained to have received the blood from one or another source.

But, the at present crowning result of these observations, is that the cruorine itself, is a sure test, for a far smaller quantity of substance by itself than either the spectroscope or micro-spectroscope can take account of, except by means of it.

If a weak solution of blood be inverted in a test tube over mercury, it reduces itself to the state of oxidation of venous cruorine, and a small prism will then show the one line spectrum, characteristic of purple cruorine, but if a single drop of distilled water be added, the oxygen in solution (not in combination) in that drop, will restore the cruorine to its scarlet state.

This change of state in the oxidized substance, the cruorine, will be at once shown in the spectroscope, but the amount of oxygen by itself which the cruorine thus appropriates, and by which it changes its state, would never be revealed *by itself*, or in any other way known to us, even by the spectroscope.

In presence of these scientific triumphs, we are impelled to inwardly exclaim, that the blood which is the life—the most wonderful of fluids, is no longer the hopeless mystery it was, but has yielded its most ulterior secrets to the patient worker.

